# Environmental Variation Contributes to Head Phenotypes in Workers of *Camponotus japonicus* (Hymenoptera: Formicidae)

**DOI:** 10.1002/ece3.71940

**Published:** 2025-08-11

**Authors:** Ruoqing Ma, Liangliang Zhang, Lv Yang, Lingxiao Tang, Xiang Zhang, Cong Wei, Hong He

**Affiliations:** ^1^ Laboratory of National Forestry and Grassland Administration for Control of Forest Biological Disasters in Western China College of Forestry, Northwest A&F University Yangling Shaanxi China; ^2^ Key Laboratory of Plant Protection Resources and Pest Management of the Ministry of Education College of Plant Protection, Northwest A&F University Yangling Shaanxi China

**Keywords:** ant, coloration, environmental variables, Gloger's rule, head phenotypic diversity, morphological traits

## Abstract

Integrating color polymorphism with intra‐species morphological traits offers substantial opportunities to study the eco‐evolutionary mechanisms underlying local population responses to heterogeneous and dynamic environments. In this study, we examined the head phenotypic diversity and intraspecific morphological traits of *Camponotus japonicus* (Mayr, 1866) workers across 22 sites in mainland China, ranging from 24°47′ N to 47°51′ N in latitude and 88°07′ E to 126°43′ E in longitude, covering an altitudinal range up to 1243 m. We classified the head phenotypes of these workers into four types and investigated environmental variables explaining the levels of polymorphism, quantified by the Shannon diversity index and head phenotype ratios. Phylogenetic analysis based on the mitochondrial *COI* gene classified all samples into four clades. By controlling the genetic distance in our analysis, we revealed the correlations between temperature, precipitation, and the prevalence and distribution of this color polymorphism across the sampled populations. Contrary to the thermal melanism hypothesis, our findings reveal that the prevalence of maroon‐headed workers (lighter coloration) increases with latitude, with the highest proportion of these individuals found in high‐latitude populations (colder, drier regions). Moreover, temperature and precipitation also show significant correlations with the morphological traits of workers, implying that specific traits may be environmentally influenced, potentially contributing to colony‐level survival in fluctuating environments. By emphasizing the often‐overlooked intraspecific variations, our research contributes to understanding how head color polymorphism and intraspecific morphological traits in ants are associated with local population responses to environmental changes.

## Graphical abstract

This study explores head color polymorphism and morphological traits in *Camponotus japonicus ants* across 22 sites in China, revealing that maroon‐headed workers (lighter color) are more common in colder, high‐latitude regions, contrary to the thermal melanism hypothesis. Temperature and precipitation significantly correlate with both color patterns and morphological traits, suggesting environmental influences on colony survival.

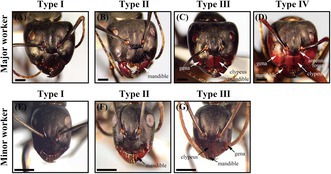

## 1 Introduction

Unraveling the factors influencing morphological variation in organisms along environmental gradients remains a persistent challenge in ecological research (Bergmann 1847; Allen 1877; Rensch 1936; Ashton 2002; Heinze et al. 2003; Classen et al. 2017; Brassard et al. 2020). Body size and body coloration are two prominent morphological traits of organisms, with coloration being a particularly complex aspect of phenotypic variation (Chown and Gaston 2010). Such variation is present at a range of geographical and ecological scales (Grant et al. 1998), driven by diverse abiotic and biotic pressures including camouflage, mimicry, sexual selection, and pathogen resistance (Clusella‐Trullas et al. 2007; Law et al. 2020). Climate, particularly temperature and precipitation, plays a crucial role in shaping the spatial distribution of organisms, exerting both direct and indirect effects on organisms (Pinkert et al. 2017; Kozlov et al. 2022). Organisms respond to these environmental influences through two primary mechanisms: genetic adaptation and phenotypic plasticity. The latter allows a genotype to produce multiple phenotypes in response to varying environmental variables, leading to modifications in behavior and physiology (Roulin 2014). Phenotypic plasticity, observed in numerous organisms in response to both biotic and abiotic environmental variables (Schlichting and Pigliucci 1998; Moczek et al. 2011; Sommer 2020), results in plastic changes in physiological and morphological traits that enhance fitness in the face of environmental variability (Agrawal et al. 1999; Miner et al. 2005; Moczek 2010).

Ecogeographical rules that associate climate with organismal form and function can illuminate the patterns of climatic association. These hypotheses used to describe clines in biological traits, such as body size and color, have typically been tested over large geographical scales through macroclimatic data (Bishop et al. 2016). For instance, Bergmann's rule states that body size increases at higher latitudes with colder climates (Bergmann 1847). In contrast, melanin‐based coloration has important functions in animals, including social communication, and is associated with various physiological, morphological, and behavioral traits (Roulin 2014). Melanin also plays a significant role in immunity and protection against UV‐B radiation, which may contribute to the adaptive significance of coloration patterns in different climates. Specifically, darker pigmentation can offer protection against UV‐B radiation, which is more prevalent in high‐altitude or high‐latitude environments with lower atmospheric shielding (Cordero and Casadevall 2020). This type of coloration is linked to climate through four key rules: Firstly, Bogert's rule, also called the thermal melanism hypothesis (TMH), predicts that animal coloration is darker in cooler environments, typically at higher latitudes or altitudes, as dark individuals absorb more solar radiation and warm up faster (Bogert 1949; Clusella‐Trullas et al. 2007). Second, the melanism‐desiccation hypothesis (MDH) predicts that darker individuals are more prevalent in drier environments, where increased melanization reduces cuticular permeability and cuticular water loss (Kalmus 1941). Third, the photoprotection hypothesis (PPH) revealed the presence of darker ants in high UV‐B environments, as the increased melanization in their cuticles is hypothesized to act as a natural sunscreen offering UV‐B protection (Bishop et al. 2016; Law et al. 2020). Last, Gloger's rule, in contrast to the pattern predicted by the TMH and MDH, suggests that organisms should have pale coloration at higher, colder latitudes, while darker colors are more prevalent in humid and warm places (Delhey 2017). The proposed mechanisms driving Gloger's rule are diverse, including camouflage, protection against parasites and pathogens, and UV‐B radiation protection (Delhey et al. 2019). Initially formulated for endotherms, Gloger's rule has been applied to a wider range of organisms (Harris et al. 2012; Bastide et al. 2014; Zheng et al. 2015).

Species with wide geographic distributions and high ecological plasticity possess the capacity to endure diverse climatic conditions and habitat types (Bellard et al. 2012). Among taxonomically diverse organisms, insects exhibit significant levels of phenotypic plasticity across various traits, encompassing morphology, pigmentation, and behavior (Braendle et al. 2006; Whitman and Ananthakrishnan 2009; Dalrymple et al. 2018). Notably, ants (Hymenoptera: Formicidae) represent one of the most abundant and highly evolved social insects in nature (Hölldobler and Wilson 1990; Passera et al. 1996). Ants are found on all continents except Antarctica and perform important ecological functions, serving as soil architects, seed dispersers, and predators (Wills and Landis 2018; Elizalde et al. 2020; Tuma et al. 2020; Parr and Bishop 2022; Schultheiss et al. 2022). Given their ubiquity and ecological significance, ants are an ideal model for investigating the complex relationship between organisms and their environment.

Exploring how ants respond to environmental conditions, particularly through their phenotypic traits, is crucial for understanding their ecological strategies. Previous research shows that temperature and precipitation significantly predict the morphological traits of ant species worldwide. In colder environments, ant colonies typically consist of larger and darker individuals (Bishop et al. 2016), whereas in warm and dry regions, they are characterized by the highest densities of polymorphic ants, where workers within a single colony exhibit notable variation in body size (La Richelière et al. 2022). This observation underscores the role of environmental conditions in driving phenotypic complexity, such as worker polymorphism (La Richelière et al. 2022). Also, darker individuals predominate in ant assemblages of the canopy, where increased melanization in their cuticles provides protection against UV‐B radiation (Law et al. 2020). These findings indicate that climate change could drive selection of ants based on cuticle color, altering community structure and potential ecosystem function. However, despite numerous studies extensively examining interspecific trait variation in ant species communities across expansive environmental gradients, comparatively little attention has been directed toward investigating the intraspecific trait variation within individual ant species (Yang et al. 2004; Ibarra‐Isassi et al. 2023; Ohyama et al. 2023; Schleuning et al. 2023). Intraspecific trait variation provides critical insights into how organisms respond to their specific environmental conditions. Notably, this intraspecific variation in ant morphology exhibits correlations with large‐scale environmental gradients, such as seasonal changes in temperature and precipitation in northern latitudes, which promote phenotypic plasticity (Brassard et al. 2020).

Understanding ant phenotypic diversity requires consideration of both genetic and environmental variables. A recent study on *Formica rufa* did not find any correlation between the variability of coloration and the kinship of workers, suggesting that environmental variables might play a more significant role than genetics in determining phenotypic traits like coloration (Skaldina and Sorvari 2020). Complementing this perspective, coloration in ants, largely determined by both genetic variation and phenotypic plasticity, serves as a response to environmental heterogeneity (Putyatina et al. 2022). Further, the study on *Formica paralugubris* suggests that although the main determinant of the facial coloration trait is genetic, environmental variables also influence its expression to some extent (Frizzi et al. 2022). This underscores that environmental variables, alongside genetic determinants, contribute to the trait's expression, albeit to varying extents. However, cuticle color as a morphological trait has mostly been studied in Formica ants because of their distinctive black‐reddish coloration and remains less explored in other ant species. Additionally, the connections between morphological traits and their ecological functions are still poorly understood, particularly at broader ecological scales. Thus, it raises a central question: does intraspecific variation in ant color adhere to predictions based on specific ecogeographic rules?

Understanding the variation in morphological traits offers profound insights into the diverse ecological strategies organisms employ to thrive under varying environmental conditions (Drager et al. 2023). Trait‐based approaches have long been integral to ecological research, with “functional traits” referring to traits that are closely linked to organism performance and ecological processes (Brousseau et al. 2018; Drager et al. 2023). In the context of ants, certain studies have identified traits as determinants of ecological function (Parr et al. 2017; Gibb et al. 2023) (refer to Table S1). The ant species *Camponotus japonicus* , with its widespread distribution in East Asia and notable characteristics such as relatively large body size and colonies, provides an excellent opportunity to study the impacts of environmental gradients on morphological traits in ants (Zhang et al. 2023; Ma et al. 2024). Moreover, *C. japonicus* displays obvious polymorphism, including queens, males, and workers, with workers further divided into “major workers” and “minor workers” based on their distinct body sizes and roles (Wheeler 1991; Smith et al. 2008). Minor workers are engaged in foraging and nursing of brood, while larger major workers primarily serve as nest defenders and rarely participate in foraging activities (Giraldo and Traniello 2014).

In this study, we conducted an examination of the head phenotype and morphological traits of *C. japonicus* workers collected from 22 distinct areas across mainland China, spanning a wide range of latitudes. To interpret the observed variation in pigmentation, we evaluated three ecogeographic hypotheses commonly applied to insect coloration: TMH, MDH, and Gloger's rule. Both the TMH and MDH predict a higher frequency of dark phenotypes in colder or drier environments. However, our results showed the opposite pattern: maroon‐headed individuals were more common in high‐latitude, colder populations. This finding is inconsistent with the TMH and MDH but aligns more closely with Gloger's rule, indicating that pigmentation in *C. japonicus* may be influenced by climatic factors beyond thermoregulation or desiccation resistance.

## 2 Materials and Methods

### 2.1 Study Sites

**FIGURE 1:**
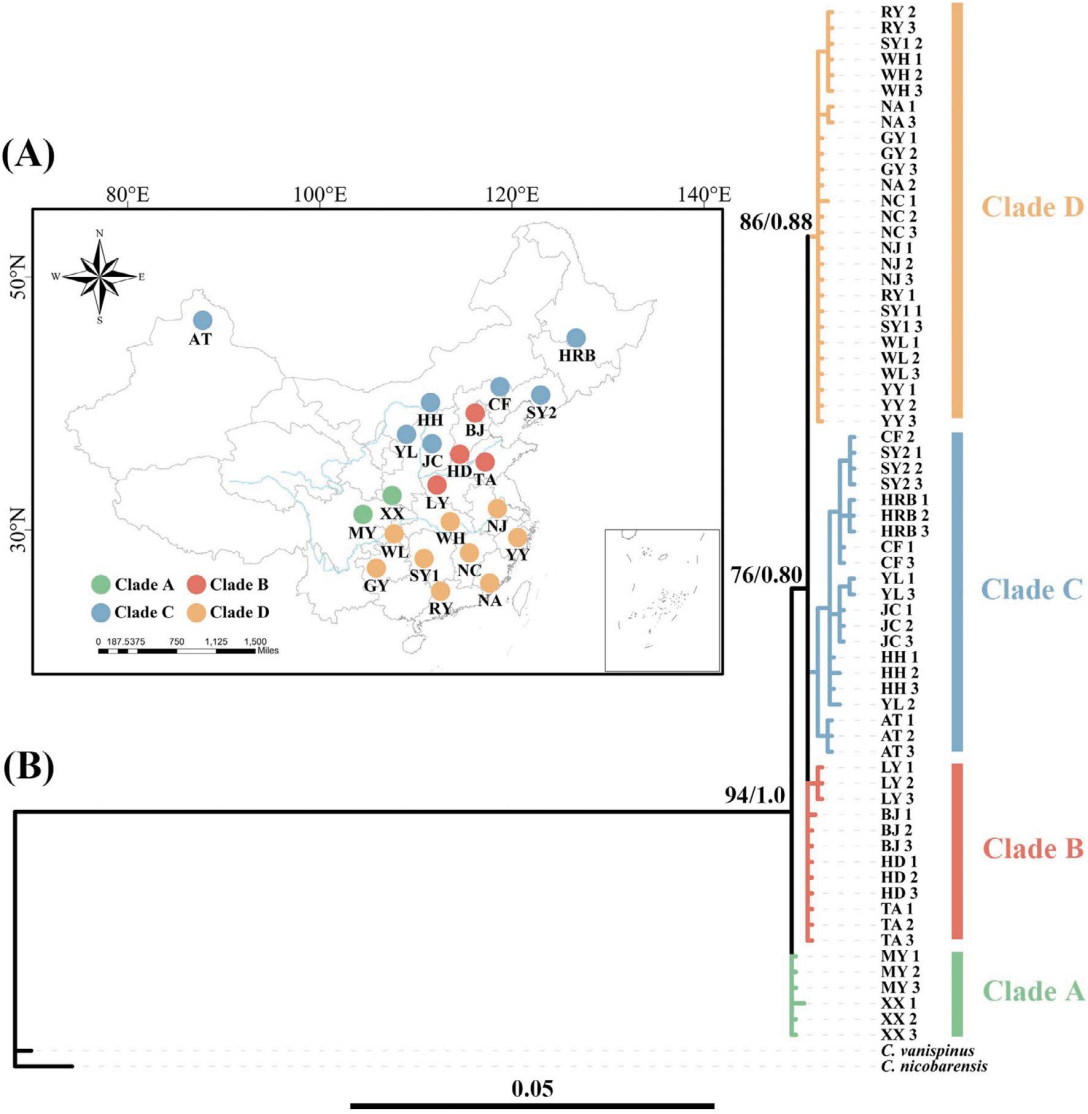
Geographic and phylogenetic representation of *C. japonicus* . (A) The geographic distribution of *C. japonicus* across various sites. Each color represents a different clade. (B) Phylogram of *C. japonicus* as inferred through maximum‐likelihood and Bayesian inference analysis for the *COI* dataset. Major clades are indicated by ML bootstrap values and Bayesian posterior probabilities near the branches.

The workers of *C. japonicus* were collected from 22 distinct sites in mainland China between April and August 2021 (Table 1, Figure 1A). The sampling sites encompassed an approximate latitudinal range of 23° (from 24° to 47° N) and an approximate longitudinal range of 38° (from 88° to 126° E). The sampled areas exhibited a mean temperature range of 21.52°C (southern region) to −0.65°C (northern region) and a mean precipitation range of 191.35 mm (southern region) to 27.75 mm (northern region). These sites encompassed the majority of the distribution range of *C. japonicus* in mainland China.

**TABLE 1:** Sample data from 22 sites of *C. japonicus* was utilized in this study.

Abbreviation	Sampling site	Longitude	Latitude	Date
AT	Altay, Xinjiang	88°07′21.62″	47°51′51.37″	July 2021
BJ	Beijing, Beijing	116°21′47.73″	39°58′31.44″	June 2021
CF	Chifeng, Neimenggu	119°0′18.36″	42°17′56.04″	July 2021
GY	Guiyang, Guizhou	106°41′56.76″	26°36′16.92″	April 2021
HD	Handan, Hebei	114°28′45.12″	36°37′48.72″	June 2021
HH	Hohhot, Neimenggu	111°37′30.31″	40°52′17.73″	July 2021
HRB	Harbin, Heilongjiang	126°43′24.96″	45°43′30.36″	August 2021
JC	Jiaocheng, Shanxi	112°08′45.96″	37°34′40.08″	May 2021
LY	Luoyang, Henan	112°27′18.86″	34°35′53.69″	May 2021
MY	Mianyang, Sichuan	104°43′35.40″	31°28′35.40″	May 2021
NA	Nanan, Fujian	118°27′35.64″	25°05′36.96″	April 2021
NC	Nanchang, Jiangxi	115°45′56.52″	28°46′37.92″	May 2021
NJ	Nanjing, Jiangsu	118°51′35.28″	32°04′33.96″	May 2021
RY	Ruyuan, Guangdong	113°14′13.92″	24°47′18.60″	April 2021
SY1	Shaoyang, Hunan	111°28′5.95″	27°12′26.84″	May 2021
SY2	Shenyang, Liaoning	123°27′50.52″	41°50′41.64″	July 2021
TA	Taian, Shandong	117°09′57.26″	36°13′35.86″	August 2021
WH	Wuhan, Hubei	114°26′51.36″	30°33′52.56″	August 0.2021
WL	Wulong, Chongqing	107°46′3.00″	29°19′55.56″	August 2021
XX	Xixiang, Shannxi	107°45′58.32″	32°59′4.92″	April 2021
YL	Yulin, Shannxi	109°43′15.75″	38°19′48.43″	April 2021
YY	Yuyao, Zhejiang	121°05′35.88″	29°43′50.52″	June 2021

### 2.2 Field Sampling of Ants

The foraging workers were manually collected at one‐hour intervals from 11 am to 5 pm within a 30‐m radius of the nest entrance during sunny weather conditions. The decision to focus on foraging workers is based on the consideration of ant colony behavior patterns, where foragers represent the most frequent interface between the colony and the external environment. Although major workers primarily serve as nest defenders and rarely forage, in this study, major workers were sampled only when observed actively foraging, ensuring that the sampled major workers represent the foraging subset. At each sampling site, foraging workers were collected from three colonies, with a separation distance of 80 to 100 m between nest entrances to prevent overlap of their foraging areas. This specific distance is based on studies showing that the foraging range of *Camponotus* species, such as *C. leydigi* and *C. sericeiventris* , can extend up to 30 m from the nest (Yamamoto and Del‐Claro 2008; Soares Jr and Oliveira 2021). Additionally, this distance was supported by observations in which *C. japonicus* workers exhibit aggressive behaviors toward non‐nestmates. Such aggression indicates distinct foraging ranges and supports the need for a clear spatial separation during sampling.

Previous studies have shown that, within ant species, worker size is positively associated with colony size—smaller workers (nanitics) are produced in the early stages, while polymorphism, including the emergence of major workers, increases as colonies mature (Brian 1957; Wood and Tschinkel 1981; Porter and Tschinkel 1985; Gibson 1989; Tschinkel 1998). Although we did not directly assess colony size in this study due to constraints in resources and field logistics, we minimized potential bias by selecting colonies that visibly contained a substantial number of major workers. This approach helps control for variation in colony development stage across populations.

Prior to molecular analysis, morphological identification of collected specimens, including *C. japonicus* , was performed using the key provided by Wang et al. (1989). Their systematic review provided the essential framework for accurate species differentiation within the *Camponotus* genus in China. The collected samples were preserved in 100% ethanol for subsequent analyses. In addition, workers of *C. nicobarensis* (Mayr, 1865) and *C. vanispinus* (Xia & Zheng, 1997) were also collected as outgroup specimens for molecular identification. Voucher specimens of the relevant ant species were deposited at the College of Forestry, Northwest A&F University (NWAFU) in Yangling City, Shaanxi Province, China.

### 2.3 Molecular Identification and Genetic Analyses

**FIGURE 2:**
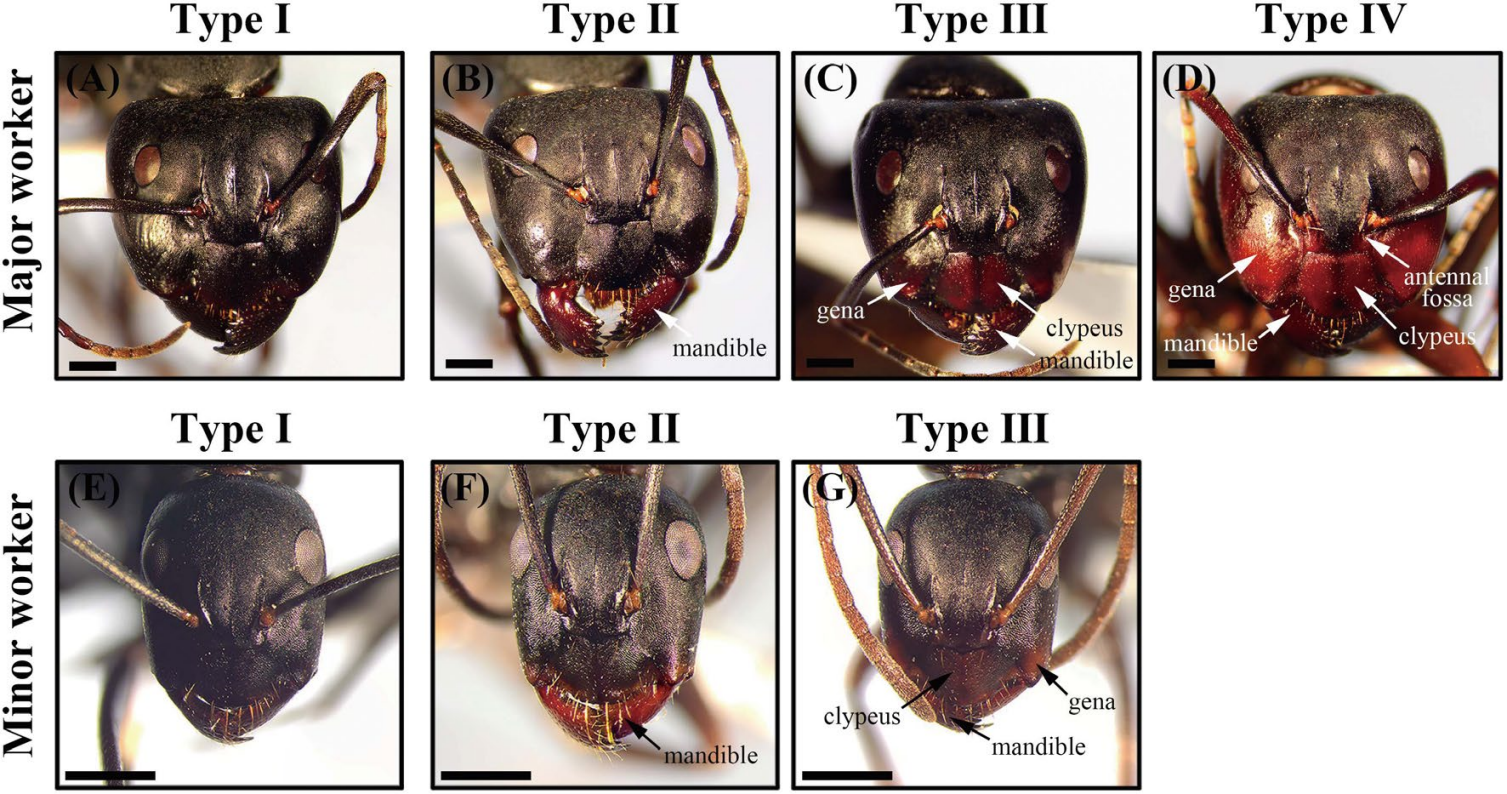
Head phenotypes of major and minor workers: Type I: Completely black; Type II: Mandible exhibits maroon coloration; Type III: Clypeus, mandible, and a limited gena region display maroon coloration; Type IV: Clypeus, mandible, antennal fossa, and a majority of the gena region exhibit vivid maroon coloration. The scale bar represents 500 μm. Photographs by Ruoqing Ma.

Three workers (including major and minor workers) were randomly selected from each nest for molecular identification to ensure the correct species for subsequent analysis. The differentiation between major and minor workers was conducted visually. This visual distinction method was chosen based on a study by Zhang (2013), which involved 1554 worker samples from 18 *C. japonicus* colonies across 18 regions of mainland China. The study revealed that minor and medium‐sized workers did not exhibit obvious morphological differences. However, major workers were clearly dimorphic compared to the smaller castes, exhibiting pronounced differences in head width and mandible length (Figure 2). Therefore, the worker caste of *C. japonicus* typically comprises two subcastes: majors and minors. Genomic DNA of ant species was extracted using the BioTeke DNA extraction kit (Beijing, China) following the manufacturer's protocol. PCR amplification of mitochondrial cytochrome oxidase I (*COI*) fragments was performed using the method described by Folmer et al. (1994), and sequencing was conducted by Tsingke (Xi'an) Biotechnology Co. Ltd. All sequences have been submitted to GenBank (Accession Numbers: *C. japonicus* : *COI*: OR398538‐OR398603. *C. nicobarensis* : *COI*: OQ266760. *C. vanispinus*: *COI*: OQ266761).

The sequences were analyzed and validated using Chromas Pro software (Technelysium Pty Ltd., Australia). Multiple sequence alignment was performed using MAFFT v.7.0 (Katoh et al. 2019). Phylogenetic analysis was conducted in PhyloSuite v.1.2.2 (Zhang et al. 2020) based on the mitochondrial *COI* gene, with the optimal nucleotide substitution model determined using ModelFinder (Kalyaanamoorthy et al. 2017). The Shimodaira‐Hasegawa‐like approximate likelihood‐ratio test (SH‐aLRT) was utilized to estimate maximum‐likelihood (ML) phylogenies using IQ‐TREE (Nguyen et al. 2015) under the HKY + F + G4 models. Additionally, 5000 ultrafast bootstraps (Minh et al. 2013) were conducted to evaluate the support for the inferred phylogenies. Bayesian Inference phylogenies were inferred using MrBayes 3.2.6 (Ronquist et al. 2012) under the HKY + F + G4 model (2 parallel runs, 2,000,000 generations), with the initial 25% of sampled data discarded as burn‐in. The topology was visualized using Figtree v.1.4.4 software (Rambaut et al. 2018). Based on the well‐supported clusters in the resulting phylogenetic trees (from both ML and Bayesian inference), we defined four major clades, each corresponding to distinct geographic groupings.

Genetic diversity assessment included the number of haplotypes (nh), haplotype diversity (hd), and nucleotide diversity (π) using DNASP. Arlequin 3.5 (Excoffier and Lischer 2010) was employed to perform *F*_ST_ calculations with 10,000 permutations. Gene flow (Nm) between the four clades was derived from *F*_ST_ values employing the formula Nm = (1 − *F*_ST_)/4 *F*_ST_.

### 2.4 Ant Head Phenotype Analyses

From the collected ant samples, 30 individuals were randomly selected per colony, including both major and minor workers, with a minimum of 10 individuals in each subcaste. Each specimen was examined under a stable lighting condition using an Olympus SZX10 stereo microscope. Images were captured using the SX60HS digital camera. The maroon color coverage proportion of major and minor workers' heads was quantified using Image J software (Schneider et al. 2012), enabling the classification of head phenotypes. Specifically, maroon pigmentation was observed on the mandibles, clypeus, antennal fossa, and gena. The classification of head phenotypes was not established a priori but was determined based on visual inspection of pigmentation patterns across individuals. We first identified distinct phenotypes according to the location of maroon pigmentation on the head capsule and then refined the classification by quantifying the proportion of maroon coverage. Based on these criteria, we defined four head phenotypes. Additionally, for each collection site, we calculated two important metrics: (1) the Shannon diversity index of the four head phenotypes, where each phenotype was treated analogously to a species in ecological diversity studies, and the relative proportion of individuals exhibiting each phenotype within a site was used as the equivalent of species abundance; and (2) the proportion of maroon‐headed workers among the total number of workers.

### 2.5 Measurement of Ant Morphological Traits

At each site, six morphological traits were measured for 30 major and 30 minor workers. From each nest within the site, 10 individuals of each subcaste were randomly selected for measurement. The following traits, namely head length (HL), head width (HW), scape length (SL), pronotum width (PW), Weber's length (WL), and mandible length (ML), were measured using the same stereo microscope and digital camera. These traits were selected because they are commonly used in ant morphological studies to characterize variation in body size, head dimensions, sensory structures, and feeding‐related morphology, thereby providing a comprehensive understanding of worker polymorphism and functional specialization. The investigation focused on examining the variations in morphological traits among major and minor worker subcastes under differing environmental conditions. The standardized protocols recommended by Parr et al. (2017), which encompass specific measurement standards for data collection, were strictly adhered to. Although additional traits such as maximum eye width were considered, they were excluded to minimize redundancy and potential multicollinearity among variables. Our measurements primarily focused on head‐associated traits that are consistently measurable and biologically relevant to head function and variation across environmental gradients.

### 2.6 Environmental Data

Six environmental variables, including annual mean temperature, annual mean precipitation, annual mean relative humidity, annual mean evapotranspiration, annual mean sunshine duration, and elevation, were integrated into the analysis (Table S2). Temperature significantly influences the physiological activities, distribution, population density, and reproductive behavior of ants (Sagata and Gibb 2016; Helms 2023; Ma et al. 2024). Precipitation, relative humidity, and evapotranspiration are direct determinants of foraging behavior and nest site selection of *C. japonicus* (Li et al. 2017; Goko et al. 2023). While some ant species are less reliant on external environmental cues due to nocturnal or subterranean habits, *C. japonicus* foraging workers in our study were consistently observed during daytime above‐ground activity, suggesting that their behavior may be more directly shaped by ambient climatic conditions. Notably, low humidity conditions deter workers from foraging due to the increased risk of dehydration and subsequent mortality (Nova et al. 2022). The consideration of sunshine duration arises from the ants' strong reliance on sunlight. It has been noted that sunshine duration has a greater impact on worker stature (Brian and Elmes 1974). Elevation plays a crucial role in shaping the distribution and behavior of *C. japonicus* by influencing temperature, humidity, and other climatic factors (Ma et al. 2024). Therefore, given the variability in elevation among sampling sites (ranging from 32 to 1275 m), we have included elevation as a factor in our analysis to address its potential indirect impacts on the species. The data for these environmental variables at the sampling sites were obtained from the NOAA‐National Centers (https://www.ncei.noaa.gov/).

### 2.7 Statistical Analysis

The Shapiro–Wilk test and the Bartlett test were conducted within the R statistical environment (R Core Team 2024) to assess the normality of distributions and the homogeneity of variances, respectively. In instances where the normality assumption was violated, non‐parametric alternatives were employed. The Kruskal‐Wallis test was employed to assess the variations in the proportion of workers with maroon heads across distinct clades, with significant intergroup differences identified through Dunn's post hoc multiple comparisons, which incorporated a Bonferroni correction.

The linear mixed model (LMM) was constructed using the “lmer” function from the “lme4” package (Bates et al. 2015). This model was developed to analyze the relationship between environmental variables (as fixed effects) and two distinct response variables: head phenotypic diversity and maroon‐headed ratios. The random effects were modeled based on the sampling location and the identity of individuals. To assess multicollinearity among the explanatory variables, the variance inflation factor (VIF) was computed using the “vif” function. Variables exhibiting a VIF exceeding 10 were omitted from the model to ensure statistical validity. The model's explanatory power was quantified using Marginal *R* ^2^ (*R*_m_^2^), representing the variance explained solely by the fixed effects, and Conditional *R* ^2^ (*R*_c_^2^), representing the variance explained by both fixed and random effects. Subsequently, the hierarchical partitioning method implemented in the “glmm. hp” function (Lai et al. 2022) was applied to quantify the individual contribution of each retained fixed effect within the model.

The interrelationships among genetic, geographic, and morphological distances for both major and minor worker subcastes were analytically assessed using the Partial Mantel test. This analysis, performed using the ‘mantel. partial’ function from the vegan package (Oksanen et al. 2023), aimed to elucidate the correlations between geographic dispersion and morphological traits while controlling for genetic distance as an indicator of neutral genetic structure. All relevant datasets, including those for geographic and morphological distances—the latter computed from Euclidean distances across six morphological traits—as well as genetic distances, underwent standardization prior to analysis.

For each sampling site, mean trait values for both major and minor workers were calculated. Preliminary analysis utilized simple linear regression to examine the relationship between these mean trait values and environmental variables, designating the mean trait values as the response variable and the environmental variables as predictors. Given altitude's significant influence on these environmental variables, a multiple linear regression analysis was employed to meticulously examine how these factors, when adjusted for altitude, affect morphological traits. This decision was informed by the observed significant impacts of temperature, precipitation, relative humidity, evapotranspiration, and altitude on morphological characteristics from the preliminary analysis. Utilizing the ‘lm’ function, we constructed a multiple linear regression model that integrates these environmental variables and altitude, with six morphological indicators serving as the dependent variables. The model's robustness was evaluated by its fit and predictive power, which was confirmed through residual analysis. For all comparisons, *p* values < 0.05 were considered statistically significant.

## 3 Results

### 3.1 Molecular Identification and Genetic Analyses

A total of 2162 workers of *C. japonicus* were collected from 22 locations across mainland China. The *COI* gene segment was successfully obtained from all samples of *C. japonicus* . Phylogenetic trees constructed using maximum‐likelihood and Bayesian inference methods exhibited a high degree of consistency. The monophyly of the *C. japonicus* samples in this study is robustly supported, as evidenced by a maximum‐likelihood bootstrap value of 94% and a Bayesian posterior probability of 1.0, which are clearly demonstrated in the phylogenetic tree (Figure 1B).

The phylogenetic analysis classified all samples into four clades (clades A, B, C, and D). Clade A is situated in the southwestern region of China, encompassing 2 sampling locations: MY and XX. Clade B is located in the central‐eastern region of China, covering 4 sampling points: BJ, HD, TA, and LY. Clade C is positioned in the northern part of China, spanning 7 sampling sites: AT, CF, HH, HRB, JC, SY2, and YL. Clade D is distributed in the southern region of China, comprising 9 sampling sites: GY, NA, NC, NJ, RY, SY1, WH, WL, and YY (Figure 1A,B). Within the four identified clades, Clade C demonstrated the highest levels of haplotype diversity (Hd = 0.914) and nucleotide diversity (π = 0.00359), followed sequentially by Clade B, Clade D, and Clade A (Table S3). The pairwise *F*_ST_ values between the four clades ranged from 0.52408 (observed between Clade B and Clade C) to 0.83458 (recorded between Clade A and Clade D), with no statistically significant differences detected among the four lineages (Table S4). Similarly, values of gene flow (Nm) exhibited variations, ranging from 0.04955 (observed between Clade A and Clade D) to 0.22703 (detected between Clade B and Clade C) (Table S4).

### 3.2 Classification and Comparison of Head Phenotypes

Although the workers of *C. japonicus* typically exhibit a blackhead phenotype, maroon‐headed workers were observed at all sampling sites. Based on the ratio of maroon and black coloration on the head of major and minor workers (Figure 2, Table 2), we identified four distinct head phenotypes. Type I represents individuals with a completely blackhead phenotype (Figure 2A,E). Type II displays maroon coloration solely on the mandible (Figure 2B,F), while Type III exhibits maroon coloration on the clypeus, mandible, and a limited gena region (Figure 2C,G). Type IV exhibits vivid maroon coloration on the clypeus, mandible, antennal fossa, and a majority of the gena region (Figure 2D). The blackhead phenotype (Type I) was the most prevalent across all sampled sites. In terms of the maroon‐head phenotype (Type II + Type III + Type IV), Type II was widely distributed across all sampling sites, while Type III was observed in most regions except for the RY (Ruyuan, Guangdong) population and NA (Nan'an, Fujian) population. Type IV was exclusively found in the major workers of the SY2 population (Shenyang, Liaoning). To quantify variation in head phenotype composition across populations, we calculated the Shannon diversity index for both major and minor workers at each sampling site (Table S5). For major workers, the highest diversity was observed in the SY2 population (1.283 ± 0.100), while the lowest occurred in the NA population (0.418 ± 0.079). A similar pattern was found in minor workers, with SY2 again exhibiting the highest diversity (1.131 ± 0.028) and NA showing the lowest (0.186 ± 0.093).

**TABLE 2:** Characteristics of head coloration types of *C. japonicus* .

Individuals identity	Type	Distribution of head colors (%)		Number of recorded individuals
		Maroon	Black	
Major worker	Type I	0–3	97–100	707
	Type II	8–16	84–92	154
	Type III	27–38	62–73	110
	Type IV	48–55	45–52	3
Minor worker	Type I	0–2	98–100	791
	Type II	12–20	80–88	107
	Type III	19–27	73–81	108

In the classification of major workers, Type I individuals were characterized by a predominant black‐head coloration, with the proportion of black color ranging from 97% to 100% and maroon present in only 0% to 3% of cases, across a dataset of 707 individuals. Conversely, Type II individuals exhibited a reduction in black coloration to 84%–92%, accompanied by an increased presence of maroon at 8%–16%, in a documented sample of 154 individuals. Further differentiation was observed in Type III individuals, where maroon coloration was elevated to 27%–38%, and black coloration correspondingly reduced to 62%–73%, within a cohort of 110 individuals. Type IV individuals demonstrated a more balanced distribution between maroon (48%–55%) and black (45%–52%) coloration, though this observation was based on a limited sample size of merely three individuals (Table 2). Among the minor worker classification, Type I individuals demonstrated a head color distribution closely aligning with that observed in their major class equivalents, characterized by a predominance of black (98%–100%) and a marginal occurrence of maroon (0%–2%), across a sample size of 791 individuals. In contrast, Type II individuals were noted for a maroon coloration proportion of 12%–20% juxtaposed with black coloration comprising 80%–88%, documented across 107 individuals. Furthermore, Type III individuals were distinguished by a maroon presence of 19%–27% against a backdrop of black ranging from 73% to 81%, within a documented sample of 108 individuals. Notably, Type IV head phenotypes were completely absent among minor workers, despite their presence in major workers of certain populations (SY2). This absence may indicate caste‐specific expression or developmental constraints associated with extensive maroon coloration.

**FIGURE 3:**
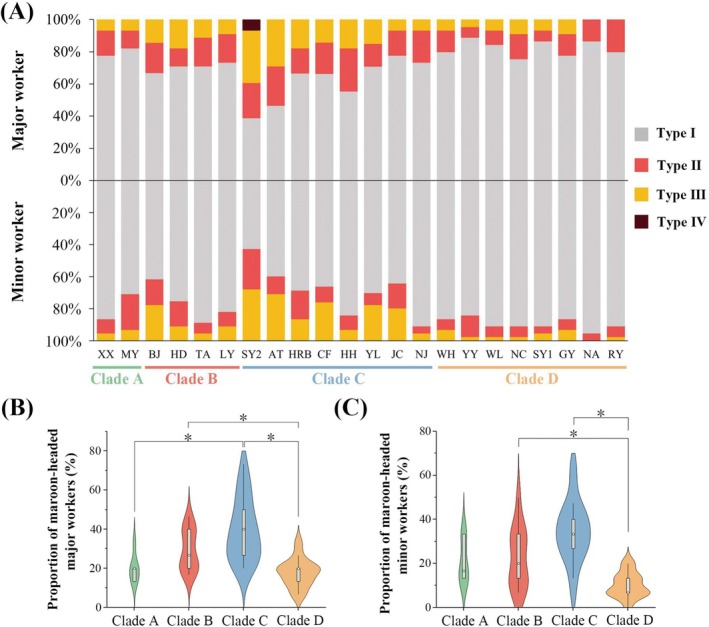
Distribution of maroon‐headed workers (Types II, III, and IV combined) in different clades. (A) Stacked bar charts represent the composition ratios of different types of maroon‐headed major (top) and minor (bottom) workers across various sampling sites. Each color represents a different coloration type (Type I–IV). (B) The proportion of maroon‐headed major workers (Types II, III, and IV combined) across four clades. (C) The proportion of maroon‐headed minor workers (Types II, III, and IV combined) across four clades. *Significant at 0.05.

Among the sampling sites, the highest proportion of maroon‐headed workers was observed in Shenyang city (SY2), Liaoning province (Northeast China), while the lowest proportion was observed in Nan'an city (NA), Fujian province (Southeast China) (Figure 3A). The Kruskal‐Wallis test indicated significant differences in the proportions of maroon‐headed workers across the different clades. For major workers, the proportions were 20.00% ± 2.98% (mean ± SE) (clade A), 29.37% ± 3.02% (clade B), 39.58% ± 3.48% (clade C), and 18.52% ± 1.30% (clade D) respectively, with a significant difference between the clades (*χ* ^2^ = 30.171, df = 3, *p* < 0.001) (Figure 3B). Subsequent post hoc comparisons using Dunn's test with Bonferroni correction revealed that the proportion in clade C was notably higher than clade A (mean difference = −3.0811, *p* = 0.0062) and clade D (mean difference = 5.2102, *p* < 0.001). Additionally, clade B showed a significantly higher proportion than clade D (mean difference = 2.7647, *p* = 0.0171). As for minor workers, the proportions were 21.11% ± 4.01% (clade A), 22.88% ± 3.93% (clade B), 34.62% ± 3.00% (clade C), and 10.12% ± 1.09% (clade D) (Figure 3C). The Kruskal‐Wallis test revealed significant differences in proportions among the clades (χ^2^ = 33.485, df = 3, *p* < 0.001). Subsequent Dunn's tests, with Bonferroni correction, indicated that clade C had a significantly higher proportion of maroon‐headed workers compared to the other three clades, particularly compared to clade D (mean difference = 5.7291, *p* < 0.001). Clade B was found to be significantly different from clade D (mean difference = 2.7301, *p* = 0.019). The proportion of maroon‐headed workers was significantly higher in clade C for both major and minor workers.

### 3.3 Head Phenotype–Environment Relationships

**FIGURE 4:**
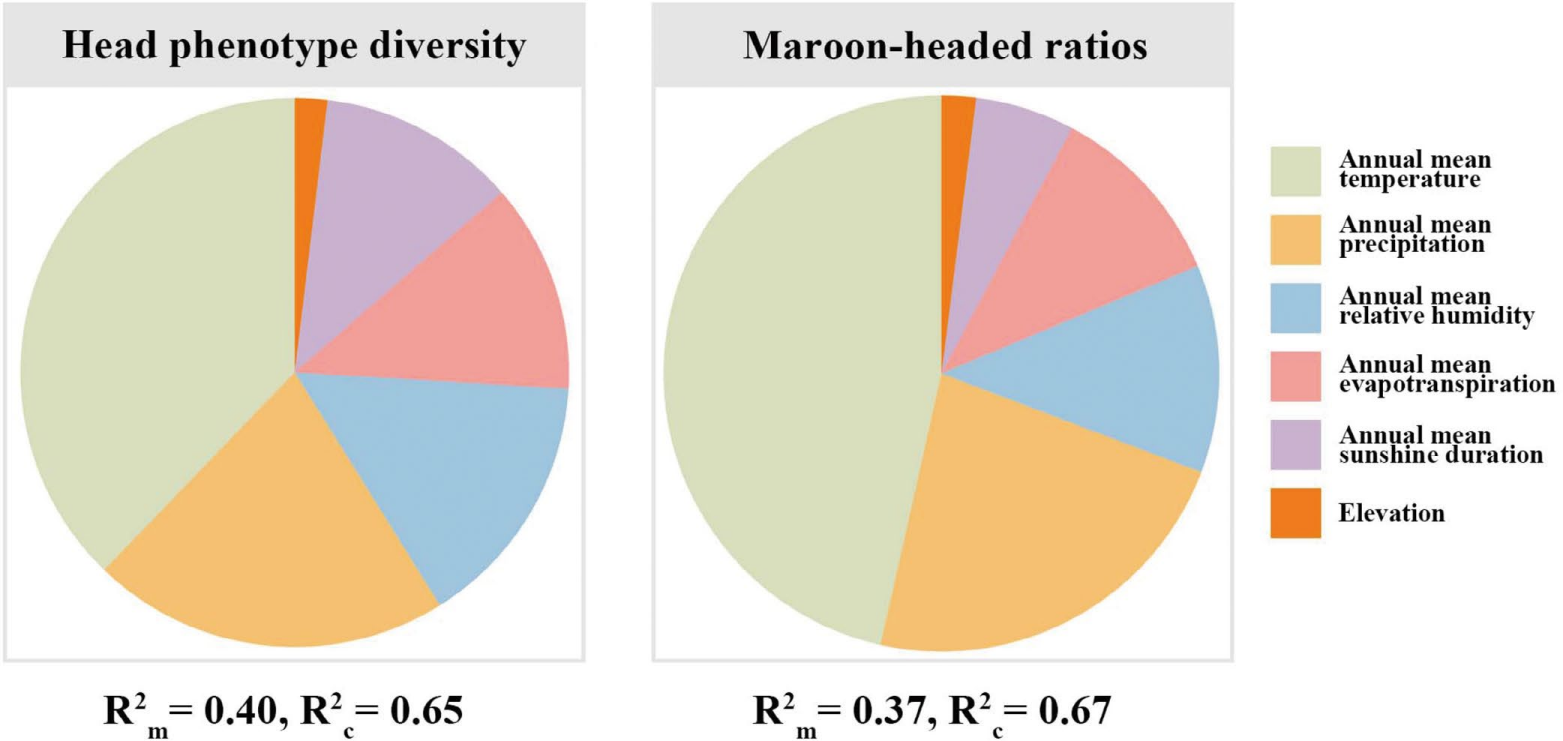
Hierarchical variance partitioning in explaining the effects of different predictor variables on head phenotypic diversity and maroon‐headed ratios. *R*_m_^2^ (marginal *R* ^2^), amount of variation that is explained by fixed factors; *R*_c_^2^ (conditional *R* ^2^), the amount of variation that is explained by both fixed and random factors.

We tested the relationship between head phenotype and environmental variables using a LMM. The fixed effects analysis within the model revealed a significant impact of temperature (*F*_(1,15)_ = 5.5653, *p* < 0.05) on head phenotypic diversity. Our model explained 65.36% (*R*_c_^2^) and 39.81% (*R*_m_^2^) of the variation in head phenotypic diversity (Figure 4, Table S6). Temperature explained 37.78% of the total variation, precipitation explained 21.10%, and relative humidity explained 15.17% (Figure 4, Table S6).

The fixed effects analysis within the model revealed significant impacts of temperature (*F*_(1,15)_ = 5.6476, *p* < 0.05) on the maroon‐headed ratios. The model explained 67.19% (*R*_c_^2^) and 37.45% (*R*_m_^2^) of the variation in maroon‐headed ratios (Figure 4, Table S6). Temperature explained 46.48% of the total variation; precipitation explained 22.74%, and relative humidity explained 12.09% (Figure 4, Table S6).

### 3.4 Trait–Environment Relationships

**FIGURE 5:**
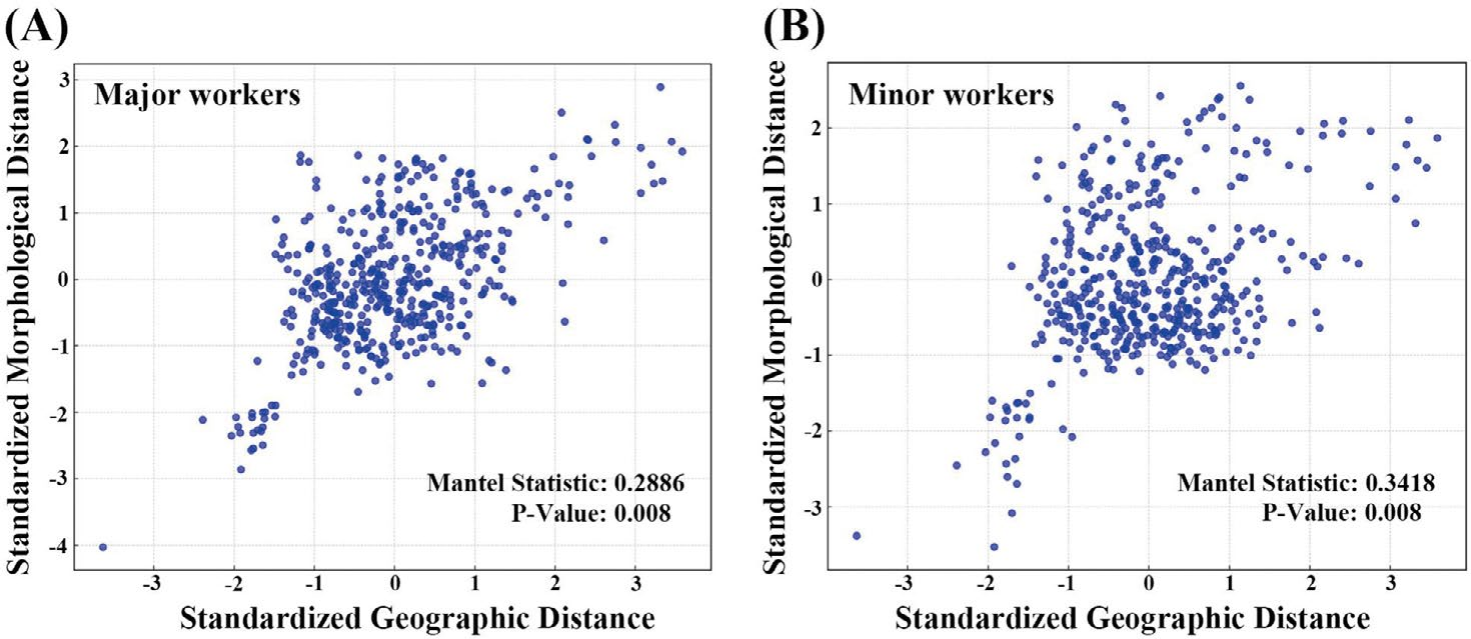
Correlation between geographic and morphological distance. (A) Major workers; (B) minor workers.

The Partial Mantel test, controlling for genetic distance as an indicator of neutral genetic structure, revealed significant correlations between geographic and morphological distances for both major and minor workers. After accounting for genetic variation, there remained a statistically significant association between the geographic distribution and morphological traits. Specifically, for major workers, the Mantel statistic (*r*) was 0.2886 (*p* < 0.01) (Figure 5A); for minor workers, it was 0.3418 (*p* < 0.01) (Figure 5B).

**FIGURE 6:**
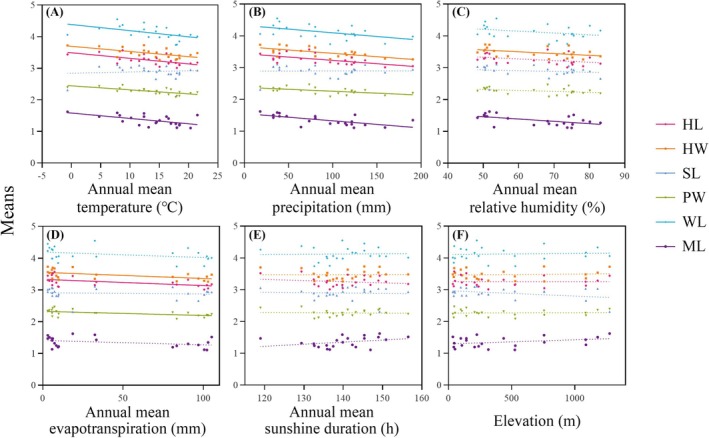
Linear regression results between traits means and environmental variables in major workers. (A) Annual mean temperature; (B) annual mean precipitation; (C) annual mean relative humidity; (D) annual mean evapotranspiration; (E) annual mean sunshine duration; and (F) elevation. Traits measured: head length (HL), head width (HW), scape length (SL), pronotum width (PW), Weber's length (WL), and mandible length (ML). Solid and dashed lines indicate significant and non‐significant trends, respectively.

**FIGURE 7:**
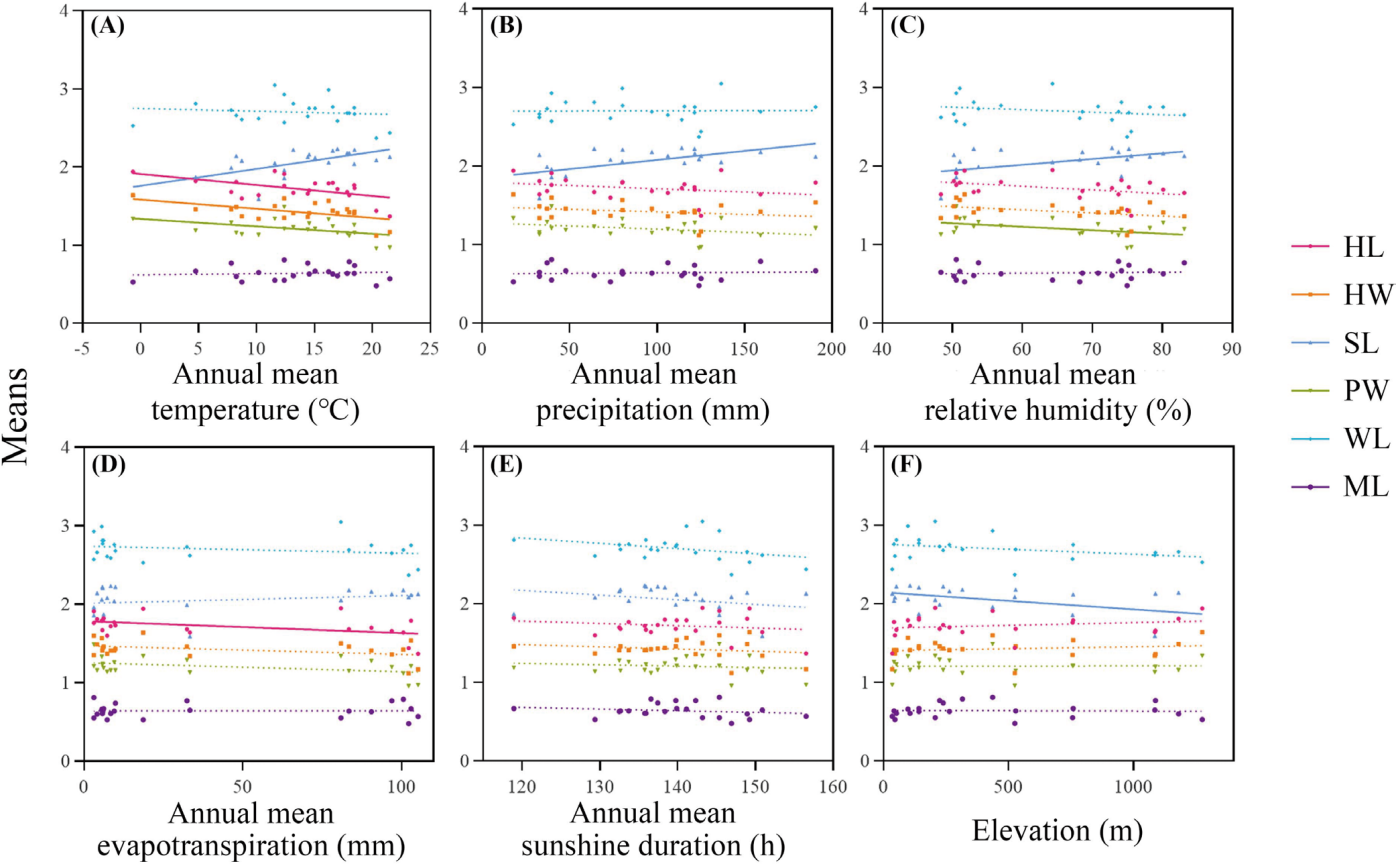
Linear regression results between traits means and environmental variables in minor workers. (A) Annual mean temperature; (B) annual mean precipitation; (C) annual mean relative humidity; (D) annual mean evapotranspiration; (E) annual mean sunshine duration; (F) elevation. Traits measured: head length (HL), head width (HW), scape length (SL), pronotum width (PW), Weber's length (WL), and mandible length (ML). Solid and dashed lines indicate significant and non‐significant trends, respectively.

The linear regression analyses revealed that the trait means of major workers exhibited significant negative correlations with temperature, precipitation, relative humidity, and evapotranspiration (Figure 6, Table S7). Specifically, both head length and pronotum width significantly decreased with increases in temperature, precipitation, and evapotranspiration. Head width showed negative correlations with relative humidity, in addition to the aforementioned variables. Weber's length was negatively correlated with temperature and precipitation, while mandible length also exhibited negative correlations with temperature, precipitation, and relative humidity. Moreover, for minor workers, both head length and head width showed significant negative correlations with temperature and evapotranspiration (Figure 7, Table S7). The scape length of minor workers was positively correlated with temperature, precipitation, and relative humidity but negatively correlated with altitude. Pronotum width decreased significantly with increasing temperature and relative humidity.

In the analysis of major workers, the multiple linear regression model demonstrated significant statistical results (*F*_(5,16)_ = 4.498, *p* < 0.05), explaining 58.43% of the variability in pronotum width (*R* ^2^ = 0.584) (Table S8). Within this analysis, among the array of environmental variables examined, a significant negative correlation was specifically observed with the annual mean temperature (β = −0.012, *t* = −2.731, *p* < 0.05). In the multiple linear regression analysis for minor workers, the model was statistically significant and revealed distinct patterns of trait associations with environmental variables. Notably, the analyses for head length (*F*_(5,16)_ = 4.767, *p* < 0.05) and head width (*F*_(5,16)_ = 3.887, *p* < 0.05) showed significant correlations with specific environmental variables (Table S9). Specifically, for head length, which exhibited an *R* ^2^ of 0.5983, a pronounced negative effect of temperature on the trait was observed (β = −0.019, *t* = −3.059, *p* < 0.05). This influence was accompanied by significant impacts from precipitation (β = 0.003, *t* = 3.072, *p* < 0.05) and evapotranspiration (β = −0.002, *t* = −2.496, *p* < 0.05), underscoring the multifaceted nature of environmental effects on morphological traits. Furthermore, the analysis concerning head width, with an *R* ^2^ of 0.5485, also pinpointed temperature (β = −0.017, *t* = −3.053, *p* < 0.05) and precipitation (β = 0.003, *t* = 2.854, *p* < 0.05) as significant predictors.

## 4 Discussion

Although commonly being referred to as “black carpenter ants,” observations across various sampling sites in mainland China reveal that *C. japonicus* workers exhibit phenotypic diversity, including a notable percentage of maroon‐headed phenotypes. In both major and minor workers, our findings indicate a higher occurrence of the maroon‐headed phenotype in colder, higher latitudes (Northern population Clade C, Figure 3A), while in warmer, lower latitudes (Southern population Clade D, Figure 3A), maroon‐headed phenotypes are less frequent, with the blackhead phenotype (Type I) predominating. This pattern is interesting, as it contradicts the TMH and MDH, aligning more closely with Gloger's rule. Although initially used in endotherms, Gloger's rule has also been widely applied to insects such as *Phaulacridium vittatum* (Harris et al. 2012), *Drosophila melanogaster* (Bastide et al. 2014), and *Sericinus montelus* (Zheng et al. 2015).

Ectotherms are sensitive to the temperature regime of their environments, where morphological traits such as body size and cuticle reflectance significantly affect heat gain and loss (Gates 1980; Pinkert et al. 2017). The TMH states that at colder, higher‐latitude environments, darker ants with more melanin are favored, since they can absorb heat more quickly (Willmer and Unwin 1981; Clusella‐Trullas et al. 2007). Indeed, studies on various ectotherms, including dragonflies, wasps, and ants, have observed that darker individuals are more often found or are more abundant in cooler environments, consistent with the TMH (Bishop et al. 2016; De Souza et al. 2017; Pinkert et al. 2017). However, our results show that in both major and minor workers, darker individuals are frequent in warm environments. This observation aligns with Gloger's rule, which posits that animal populations inhabiting warmer, wetter regions tend to exhibit darker pigmentation, whereas those in cooler, drier areas tend to be lighter in color (Delhey 2017). Similarly, studies on other insects, such as beetles, butterflies, and grasshoppers living in tropical regions, tend to have darker pigmentation than those living in more temperate regions (Sømme 1989; Mani 2013), which supports the applicability of Gloger's rule in entomological research. We also noted behavioral differences between regions: northern populations exhibited more temporally constrained foraging activity around midday, likely a response to colder temperatures, while southern populations showed a longer daily foraging window, potentially benefiting from warmer conditions. This behavioral plasticity may interact with pigmentation patterns to enhance ecological adaptation.

This color variation in the head cuticle is primarily due to the differential responses to climatic variation of both main types of melanin: the presence of brown to black eumelanin and yellow to reddish‐brown pheomelanin (Ducrest et al. 2008; Delhey et al. 2019). Chiefly, melanin deposition increases with high temperature and decreases with low temperature, whereby pheomelanin decreases first, while eumelanin only decreases with extreme cold. Dryness leads to a decrease in eumelanin and an increase in pheomelanin (Görnitz 1923; Delhey et al. 2019). This theory can well explain our results: In colder, dryer areas, eumelanin decreases and pheomelanin increases, so the proportion of maroon‐headed phenotypes is the highest in high‐latitude areas; when the temperature rises, melanin deposition increases, so the proportion of maroon‐headed phenotypes decreases in low‐latitude warm areas. Notwithstanding, opinions about the mechanism behind the change of animal color with climate are not consistent. For example, unlike Gloger, Allen concluded that humidity or rainfall was more important for animal coloration than temperature or solar radiation (Delhey et al. 2019). However, they both thought that variation in coloration due to climate was mainly a plastic response and that there were no real adaptive benefits to this variation (Delhey et al. 2019). Similarly, in our study of *C. japonicus* , where the predominant phenotype is black, the presence of maroon‐headed phenotypes can be seen as a plastic response to local environmental variables (Galeotti and Rubolini 2004; Roulin 2004; Galeotti et al. 2009). Therefore, the prevalence of reddish morphs in a given population (northern population, Clade C) may reflect adjustments to the local environment in this species, suggesting that polymorphism in ant coloration may play a significant role in their ability to occupy diverse ecological niches. Color polymorphism can be advantageous for populations and species, as it may reduce extinction risk (Forsman 2016; Kozlov et al. 2022). While existing research supports that color variation may have evolved as a response to climate change, more data is needed to confirm its prevalence in insects and to understand the mechanisms of this response (Clusella‐Trullas and Nielsen 2020).

Biotic interactions have been shown to be more intense in warm climates (Schemske et al. 2009), which might favor polymorphic phenotypes, especially in major workers involved in colony defense, through selection and social regulation (Planqué et al. 2016). Consistent with this, the Northern population, Clade C, exhibited the most diverse head phenotypes. Notably, we observed that only major workers in SY2 (Clade C) displayed type IV phenotypes, with a maroon coverage rate as high as 51.65%, displaying vivid maroon coloration. Interestingly, similar microevolutionary divergences in worker caste ratios and body sizes, reflecting different investment strategies in colony structure, have been documented in geographically distinct populations of *Pheidole morrisi* (Yang et al. 2004). Contrary to typical findings, our results emerged from a colder environment. SY2 is situated in a frigid high‐latitude area, with an average annual temperature of only 8.74°C, which is significantly lower than other locations. A similar trend was observed in high‐latitude leaf beetles; the highest level of color polymorphism was also found at high latitude (Kozlov et al. 2022). Furthermore, Clade C showed the highest haplotype and nucleotide diversity, suggesting that ant populations in northern China may have experienced heightened gene exchange or intensified selective pressures (Clusella‐Trullas and Nielsen 2020). While climate‐related color variation may often reflect enhanced fitness of certain phenotypes, it can also arise from phenotypic plasticity or be shaped by other selection forces such as predation or gene flow (Clusella‐Trullas and Nielsen 2020). In some insects, high‐contrast color patterns may serve as disruptive coloration that reduces detectability by predators, but it remains unclear whether similar mechanisms occur in ants (Cuthill et al. 2005).

Our study reveals that temperature and precipitation explained a substantial portion of the diversity in head phenotypes and maroon‐headed ratios in *C. japonicus* (Figure 5, Table S6); both factors negatively correlated with these variations. These environmental variables are recognized as key drivers shaping animal phenotypes (Pinkert et al. 2017; Brassard et al. 2020; Kozlov et al. 2022; La Richelière et al. 2022). This suggests that cold and dry environments promote the occurrence of maroon‐headed workers, a pattern inconsistent with the TMH and MDH but more aligned with Gloger's rule, which predicts lighter coloration in colder and drier regions. The unexpected prevalence of maroon‐headed individuals under these conditions implies that pigmentation may serve functions beyond thermoregulation and water conservation. Melanin contributes to UV protection and immune defense (Roulin 2014; Cordero and Casadevall 2020), and the reddish (maroon) phenotypes may reflect a balance between eumelanin and pheomelanin production responding to climatic pressures. In high‐latitude regions, increased UV exposure due to thinner atmospheric shielding may favor lighter or reddish pigmentation that offers UV protection while minimizing physiological costs. Interestingly, while the scape length of major workers did not show significant correlations with environmental variables, the scape length of minor workers was significantly correlated with temperature, precipitation, relative humidity, and altitude. This variation may reflect not only the division of labor within the colony, as minor workers are primarily responsible for foraging and brood care and thus benefit from elongated antennae for enhanced chemical detection (Weiser and Kaspari 2006; Giraldo and Traniello 2014; Yates et al. 2014), but also a pattern consistent with Allen's rule. According to this ecogeographical principle, animals in warmer environments tend to evolve longer appendages to enhance heat dissipation (Allen 1877; Bidau and Marti 2008). Although originally proposed for endothermic vertebrates, this rule has also been supported in various insect taxa, suggesting its broader applicability to morphological adaptation. This variation in body size influences colony dynamics by affecting survival, competitive ability, and reproduction through strategic investment in different castes (Whitehouse and Jaffe 1996; Powell 2009).

In conclusion, we investigated the intraspecific variation of *C. japonicus* across environmental gradients, focusing on the variability of the head coloration and other morphological traits among different populations. Our observations revealed that both phenotypic diversity and the proportion of maroon‐headed individuals were higher in the colder and drier high latitudes. This finding diverges from the predictions of the TMH and aligns more closely with Gloger's rule. Our study demonstrates that coloration differences are correlated with environmental variables, notably temperature and precipitation. Among these variables, temperature appeared to be the most influential. These insights contribute to our understanding of the potential factors leading to evolutionary dynamics that influence phenotypic diversity in response to environmental variations. Further studies are required to determine whether this color variation provides adaptive benefits like camouflage or photoprotection.

## Author Contributions

**Ruoqing Ma:** conceptualization (lead), investigation (lead), methodology (lead), resources (equal), writing – original draft (equal). **Liangliang Zhang:** conceptualization (equal), formal analysis (equal), investigation (equal), methodology (equal), writing – original draft (equal). **Lv Yang:** investigation (equal). **Lingxiao Tang:** software (equal). **Xiang Zhang:** software (equal). **Cong Wei:** resources (equal), writing – review and editing (equal). **Hong He:** funding acquisition (lead), writing – review and editing (lead).

## Conflicts of Interest

The authors declare no conflicts of interest.

## Supporting information


**Table S1:** Description of the ant traits examined in this study and their hypothesized functional response.
**Table S2:** Environmental factors of each sampling site.
**Table S3:** Genetic diversity calculated for four clades.
**Table S4:** Pairwise genetic differentiation (*F*_ST_ in lower diagonal) and gene flow (Nm in upper diagonal) between different clades of *C. japonicus* .
**Table S5:** Shannon diversity index (head phenotypic diversity) of major and minor workers across sampling sites.
**Table S6:** Hierarchical variance partitioning in driving the effects of variables on head phenotype diversity and maroon‐headed ratios.
**Table S7:** Linear regression estimates of trait means across worker subcaste and environmental variables. Significant values are highlighted in bold.
**Table S8:** Multiple linear regression estimates of trait means across worker subcaste and environmental variables. Significant values are highlighted in bold.
**Table S9:** Multiple linear regression estimates of trait means across worker subcaste and environmental variables. Significant values are highlighted in bold.

## Data Availability

All the required data are uploaded as Supporting Information.
