# Supplementary material for: Environmental Variation Contributes to Head Phenotypes in Workers of Camponotus japonicus (Hymenoptera: Formicidae)
Source: Ecol Evol. 2025 Aug 11;15(8):e71940. doi: 10.1002/ece3.71940 (PMC12336421; doi:10.1002/ece3.71940)
Supplement: Supplementary file 1 — Table S1: Description of the ant traits examined in this study and their hypothesized functional response. Table S2: Environmental factors of each sampling site. Table S3: Genetic diversity calculated for four clades. Table S4: Pairwise genetic differentiation (F ST in lower diagonal) and gene flow (Nm in upper diagonal) between different clades of C. japonicus . Table S5: Shannon diversity index (head phenotypic diversity) of major and minor workers across sampling sites. Table S6: Hierarchical variance partitioning in driving the effects of variables on head phenotype diversity and maroon‐headed ratios. Table S7: Linear regression estimates of trait means across worker subcaste and environmental variables. Significant values are highlighted in bold. Table S8: Multiple linear regression estimates of trait means across worker subcaste and environmental variables. Significant values are highlighted in bold. Table S9: Multiple linear regression estimates of trait means across worker subcaste and environmental variables. Significant values are highlighted in bold. [file ECE3-15-e71940-s001.docx]

**Appendices**

**Tab. S1:** Description of the ant traits examined in this study and their hypothesized functional response. All measurements are in millimeters (mm). The illustrations of different parts of ant bodies were modified from Parr & al. (2017).

| **Trait** | **Illustration** | **Measure** | **Hypothesized function** | **Source** |
| --- | --- | --- | --- | --- |
| Head length (HL) | 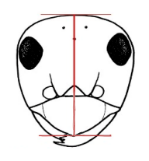 | Maximum vertical length in full face view | Relates to diet, longer head length may indicate herbivory | Kaspari 1993; Sarty & al. 2006 |
| Head width (HW) | 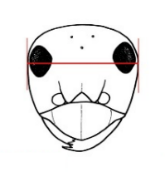 | Maximum horizontal length in full face view (across eyes) | Relates to mandible strength and predatory strategies | Kaspari 1993; Sarty & al. 2006 |
| Scape length (SL) |  | Maximum length of first antenna segment in a straight line | Relates to chemical cue sensory abilities. Longer scape facilitates pheromone trail following | Weiser & Kaspari 2006; Yates & al. 2014 |
| Pronotum width (PW) | 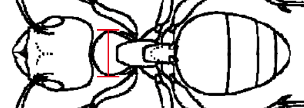 | Pronotum longitudinal length in dorsal view | Indicative of body size and often linked with resource use and habitat maneuverability | Wiernasz & Cole 2003; Sarty & al. 2006 |
| Weber's length (WL) |  | Maximum length measured from the anterior edge of pronotum to posterior edge of propodeum | Associated with food size, foraging preference and defense strategies. | Weber 1938; Kaspari & Weiser 1999 |
| Mandible length (ML) |  | Maximum straight-line from mandibular apex to anterior clypeal margin in full face view | Mandible size relates to predatory behaviour, with larger mandibles allowing for larger prey capture. | Weiser & Kaspari 2006; Gibb & Cunningham 2013 |

Gibb, H., and S. A. Cunningham. 2013. “Restoration of Trophic Structure in an Assemblage of Omnivores, Considering a Revegetation Chronosequence.” *Journal of Applied Ecology* 50, no. 2: 449–458.

Kaspari, M. 1993. “Body Size and Microclimate Use in Neotropical Granivorous Ants.” *Oecologia* 96: 500–507.

Kaspari, M., and M. D. Weiser. 1999. “The Size–Grain Hypothesis and Interspecific Scaling in Ants.” *Functional Ecology* 13, no. 4: 530–538.

Sarty, M., K. L. Abbott, and P. J. Lester. 2006. “Habitat Complexity Facilitates Coexistence in a Tropical Ant Community.” *Oecologia* 149: 465–473.

Weber, N. A. 1938. “The Biology of the Fungus‐Growing Ants. Part IV. Additional New Forms. Part V. The Attini of Bolivia. Biología de Las Hormigas Cultivadoras de Hongos. Parte IV. Nuevas Formas Adicionales. Part V. Las Attini de Bolivia.” *Revista De Entomologia* 9: 154–206.

Wiernasz, D. C., and B. J. Cole. 2003. “Queen Size Mediates Queen Survival and Colony Fitness in Harvester Ants.” *Evolution* 57, no. 9: 2179–2183.

**Tab. S2:** Environmental factors of each sampling site.

| **Abbreviation** | **Annual mean temperature (°C)** | **Annual mean precipitation (mm)** | **Annual mean relative humidity (%)** | **Annual mean evapotranspiration (mm)** | **Annual mean sunshine duration (h)** | **Elevation (m)** |
| --- | --- | --- | --- | --- | --- | --- |
| **AT** | -0.65 | 17.85 | 51.74 | 18.63 | 149.23 | 1275 |
| **BJ** | 13.18 | 64.00 | 53.07 | 5.83 | 135.93 | 52 |
| **CF** | 7.84 | 37.16 | 53.71 | 32.40 | 139.76 | 224 |
| **GY** | 14.42 | 106.11 | 83.14 | 96.93 | 142.30 | 1088 |
| **HD** | 16.22 | 80.09 | 51.06 | 5.67 | 141.13 | 98 |
| **HH** | 8.27 | 32.71 | 50.22 | 4.11 | 148.95 | 1180 |
| **HRB** | 4.75 | 47.82 | 74.14 | 6.23 | 118.94 | 141 |
| **JC** | 12.39 | 39.61 | 50.55 | 3.10 | 145.38 | 756 |
| **LY** | 16.58 | 80.36 | 56.94 | 5.90 | 138.34 | 142 |
| **MY** | 17.84 | 96.99 | 68.57 | 83.41 | 132.63 | 529 |
| **NA** | 21.52 | 125.13 | 75.56 | 105.25 | 156.50 | 35 |
| **NC** | 18.02 | 159.11 | 73.73 | 100.54 | 136.57 | 316 |
| **NJ** | 16.98 | 114.17 | 72.82 | 8.44 | 135.78 | 106 |
| **RY** | 20.34 | 123.99 | 74.98 | 102.22 | 146.92 | 525 |
| **SY1** | 18.47 | 121.33 | 75.22 | 9.94 | 137.43 | 239 |
| **SY2** | 8.74 | 73.43 | 68.20 | 7.33 | 129.39 | 46 |
| **TA** | 11.59 | 136.63 | 64.30 | 80.92 | 143.19 | 207 |
| **WH** | 18.43 | 115.63 | 70.80 | 9.58 | 133.70 | 42 |
| **WL** | 14.55 | 121.51 | 80.12 | 90.48 | 132.48 | 264 |
| **XX** | 12.39 | 39.61 | 50.55 | 3.10 | 145.38 | 436 |
| **YL** | 10.18 | 32.75 | 48.37 | 33.35 | 150.90 | 1086 |
| **YY** | 15.07 | 190.53 | 78.21 | 102.99 | 139.87 | 759 |

Note: Data is obtained from NOAA-National Centers for Environmental Information.

**Tab. S3:** Genetic diversity calculated for four clades.

| **Clade** | **Number of haplotypes** | **Haplotype diversity (Hd)** | **Nucleotide diversity (π)** |
| --- | --- | --- | --- |
| **Clade A** | 2 | 0.333 | 0.00101 |
| **Clade B** | 3 | 0.530 | 0.00088 |
| **Clade C** | 9 | 0.914 | 0.00359 |
| **Clade D** | 4 | 0.519 | 0.00087 |

**Tab. S4:** Pairwise genetic differentiation (*F*_ST_ in lower diagonal) and gene flow (Nm in above diagonal) between different clades of *C. japonicus*.

| **Clade** | **Clade A** | **Clade B** | **Clade C** | **Clade D** |
| --- | --- | --- | --- | --- |
| **Clade A** | – | 0.07471 | 0.15282 | 0.04955 |
| **Clade B** | 0.76992 | – | 0.22703 | 0.13188 |
| **Clade C** | 0.62063 | 0.52408 | – | 0.11425 |
| **Clade D** | 0.83458 | 0.65465 | 0.68635 | – |

**Tab. S5:** Shannon diversity index (Head phenotypic diversity) of major and minor workers across sampling sites.

| **Abbreviation** | **Major (Mean ± SE)** | **Minor (Mean ± SE)** |
| --- | --- | --- |
| **AT** | 1.097 ± 0.043 | 0.970 ± 0.025 |
| **BJ** | 0.896 ± 0.128 | 0.971 ± 0.061 |
| **CF** | 0.932 ± 0.048 | 0.830 ± 0.125 |
| **GY** | 0.740 ± 0.046 | 0.508 ± 0.122 |
| **HD** | 0.836 ± 0.095 | 0.751 ± 0.100 |
| **HH** | 1.007 ± 0.094 | 0.554 ± 0.160 |
| **HRB** | 0.918 ± 0.047 | 0.876 ± 0.028 |
| **JC** | 0.675 ± 0.076 | 0.931 ± 0.036 |
| **LY** | 0.794 ± 0.100 | 0.544 ± 0.196 |
| **MY** | 0.647 ± 0.048 | 0.777 ± 0.123 |
| **NA** | 0.418 ± 0.079 | 0.186 ± 0.093 |
| **NC** | 0.772 ± 0.078 | 0.324 ± 0.202 |
| **NJ** | 0.787 ± 0.051 | 0.369 ± 0.091 |
| **RY** | 0.534 ± 0.000 | 0.328 ± 0.049 |
| **SY1** | 0.508 ± 0.122 | 0.328 ± 0.049 |
| **SY2** | 1.283 ± 0.100 | 1.131 ± 0.028 |
| **TA** | 0.840 ± 0.087 | 0.461 ± 0.091 |
| **WH** | 0.694 ± 0.000 | 0.508 ± 0.122 |
| **WL** | 0.599 ± 0.048 | 0.369 ± 0.091 |
| **XX** | 0.663 ± 0.129 | 0.510 ± 0.042 |
| **YL** | 0.854 ± 0.093 | 0.773 ± 0.122 |
| **YY** | 0.461 ± 0.091 | 0.504 ± 0.039 |

**Tab. S6:** Hierarchical variance partitioning in driving the effects of variables on head phenotype diversity and maroon-headed ratios.

|  | **Variable** | **Unique** | **Average.share** | **Individual** | **Perc (%)** |
| --- | --- | --- | --- | --- | --- |
| **H****ead phenotype diversity** | Annual mean temperature | 0.0476 | 0.1028 | 0.1504 | 37.78 |
|  | Annual mean precipitation | -0.0015 | 0.0855 | 0.084 | 21.1 |
|  | Annual mean relative humidity | 0.0199 | 0.0405 | 0.0604 | 15.17 |
|  | Annual mean evapotranspiration | -0.0033 | 0.0499 | 0.0466 | 11.71 |
|  | Annual mean sunshine duration | 0.0321 | 0.017 | 0.0491 | 12.33 |
|  | Elevation | -0.0042 | 0.0118 | 0.0076 | 1.91 |
| **M****aroon-headed ratios** | Annual mean temperature | 0.0759 | 0.0982 | 0.1741 | 46.48 |
|  | Annual mean precipitation | -0.0029 | 0.0881 | 0.0852 | 22.74 |
|  | Annual mean relative humidity | 0.0000 | 0.0453 | 0.0453 | 12.09 |
|  | Annual mean evapotranspiration | -0.0066 | 0.0474 | 0.0408 | 10.89 |
|  | Annual mean sunshine duration | 0.0009 | 0.0208 | 0.0217 | 5.79 |
|  | Elevation | -0.0012 | 0.0087 | 0.0075 | 2 |

**Tab. S7:** Linear regression estimates of trait means across worker subcaste and environmental variables. Significant values are highlighted in bold.

| **Worker subcaste** | | **Major worker** | | | | | | **Minor worker** | | | | | |
| --- | --- | --- | --- | --- | --- | --- | --- | --- | --- | --- | --- | --- | --- |
| **Trait** | | **HL** | **HW** | **SL** | **PW** | **WL** | **ML** | **HL** | **HW** | **SL** | **PW** | **WL** | **ML** |
| **Annual Mean Temperature** | *R^2^* | **0.3725** | **0.3557** | 0.0136 | **0.4615** | **0.2441** | **0.3266** | **0.2913** | **0.2762** | **0.4399** | **0.1899** | 0.0140 | 0.0085 |
|  | *Slope* | **-0.0176** | **-0.0164** | 0.0039 | **-0.0128** | **-0.0195** | **-0.0170** | **-0.0141** | **-0.0117** | **0.0217** | **-0.0096** | -0.0036 | 0.0016 |
|  | *t* | **-3.4535** | **-3.3124** | 0.5289 | **-4.1886** | **-2.5264** | **-3.1383** | **-2.7983** | **-2.7759** | **3.9631** | **-2.1970** | -0.5365 | 0.4245 |
|  | *p* | **0.0026** | **0.0034** | 0.6049 | **0.0005** | **0.0194** | **0.0055** | **0.0095** | **0.0120** | **0.0008** | **0.0426** | 0.6003 | 0.6827 |
| **Annual Mean Precipitation** | *R^2^* | **0.3912** | **0.4411** | 0.0001 | **0.3133** | **0.2592** | **0.4293** | 0.0773 | 0.0629 | **0.3685** | 0.1044 | 0.0002 | 0.0038 |
|  | *Slope* | **-0.0021** | **-0.0021** | 0.0001 | **-0.0012** | **-0.0023** | **-0.0023** | -0.0008 | -0.0006 | **0.0022** | -0.0008 | 0.0000 | 0.0001 |
|  | *t* | **-3.6022** | **-3.9501** | 0.0330 | **-3.0334** | **-2.6403** | **-3.9195** | -1.2820 | -1.1808 | **3.4166** | -1.5464 | 0.0434 | 0.2805 |
|  | *p* | **0.0019** | **0.0007** | 0.9784 | **0.0068** | **0.0155** | **0.0009** | 0.2102 | 0.2605 | **0.0027** | 0.1424 | 0.9496 | 0.7839 |
| **Annual Mean relative humidity** | *R^2^* | 0.1553 | **0.2024** | 0.0258 | 0.1270 | 0.1041 | **0.2914** | 0.1575 | 0.1558 | **0.2406** | **0.1972** | 0.0546 | 0.0062 |
|  | *Slope* | -0.0052 | **-0.0056** | -0.0024 | -0.0031 | -0.0058 | **-0.0073** | -0.0047 | -0.0040 | **0.0073** | **-0.0044** | -0.0032 | 0.0006 |
|  | *t* | -1.9191 | **-2.2493** | -0.7195 | -1.7162 | -1.5247 | **-2.9110** | -1.9226 | -1.9488 | **2.5172** | **-2.2522** | -1.1110 | 0.3158 |
|  | *p* | 0.0695 | **0.0356** | 0.4755 | 0.1035 | 0.1431 | **0.0095** | 0.0675 | 0.0691 | **0.0205** | **0.0384** | 0.2952 | 0.7274 |
| **Annual Mean evapotranspiration** | *R^2^* | **0.2930** | **0.3102** | 0.0096 | **0.2970** | 0.1225 | 0.1334 | **0.2115** | 0.1554 | 0.0583 | 0.1701 | 0.0563 | 0.0000 |
|  | *Slope* | **-0.0020** | **-0.0019** | -0.0004 | **-0.0013** | -0.0017 | -0.0014 | **-0.0015** | -0.0011 | 0.0010 | -0.0011 | -0.0009 | 0.0000 |
|  | *t* | **-2.8789** | **-3.0020** | -0.4147 | **-2.9252** | -1.6750 | -1.7550 | **-2.3098** | -1.9527 | 1.1131 | -2.0115 | -1.0981 | 0.0437 |
|  | *p* | **0.0093** | **0.0071** | 0.6637 | **0.0087** | 0.1103 | 0.0947 | **0.0313** | 0.0694 | 0.2789 | 0.0565 | 0.2878 | 0.9735 |
| **Annual Mean sunshine duration** | *R^2^* | 0.0367 | 0.0001 | 0.0037 | 0.0037 | 0.0006 | 0.1149 | 0.0299 | 0.0367 | 0.0781 | 0.0150 | 0.1154 | 0.0362 |
|  | *Slope* | -0.0036 | 0.0001 | -0.0013 | -0.0007 | 0.0007 | 0.0066 | -0.0029 | -0.0028 | -0.0059 | -0.0017 | -0.0066 | -0.0021 |
|  | *t* | -0.8719 | 0.0285 | -0.2405 | -0.2922 | 0.0992 | 1.6287 | -0.7665 | -0.8418 | -1.3017 | -0.5351 | -1.6015 | -0.8422 |
|  | *p* | 0.3929 | 0.9757 | 0.7893 | 0.7874 | 0.9105 | 0.1228 | 0.4418 | 0.3929 | 0.2078 | 0.5877 | 0.1219 | 0.3967 |
| **Elevation** | *R^2^* | 0.0001 | 0.0075 | 0.1463 | 0.0020 | 0.0034 | 0.1096 | 0.0409 | 0.0285 | **0.2547** | 0.0003 | 0.1015 | 0.0012 |
|  | *Slope* | 0.0000 | 0.0000 | -0.0002 | 0.0000 | 0.0000 | 0.0001 | 0.0000 | 0.0000 | **-0.0002** | 0.0000 | -0.0001 | 0.0000 |
|  | *t* | -0.0134 | 0.4077 | -1.8282 | 0.1837 | 0.2576 | 1.5682 | 0.9162 | 0.7653 | **-2.6143** | 0.0764 | -1.4999 | -0.1611 |
|  | *p* | 0.9871 | 0.7019 | 0.0790 | 0.8430 | 0.7962 | 0.1323 | 0.3667 | 0.4528 | **0.0166** | 0.9429 | 0.1485 | 0.8768 |

**Tab. S8:** Multiple linear regression estimates of trait means across worker subcaste and environmental variables. Significant values are highlighted in bold.

| **Worker subcaste** | **Trait** | **R^2^** | **Independent variable** | **Unstandardized coefficient** | | **Standardized coefficient** | ***t*** | ***P* value** |
| --- | --- | --- | --- | --- | --- | --- | --- | --- |
|  |  |  |  | **B** | **Std. error** | **Beta** |  |  |
| **Major worker** | **HL** | 0.5544 | Intercept | 3.4777 | 0.2166 |  | 16.0526 | <0.05 |
|  |  |  | Temperature | -0.0128 | 0.0071 | -0.4445 | -1.7954 | 0.0915 |
|  |  |  | Precipitation | -0.0016 | 0.0012 | -0.4673 | -1.2909 | 0.2151 |
|  |  |  | Relative humidity | 0.0023 | 0.0036 | 0.1771 | 0.6435 | 0.5290 |
|  |  |  | Evapotranspiration | -0.0004 | 0.0010 | -0.1100 | -0.4023 | 0.6928 |
|  |  |  | Elevation | -0.0001 | 0.0001 | -0.2855 | -1.3406 | 0.1988 |
|  | **HW** | 0.5274 | Intercept | 3.6645 | 0.2123 |  | 17.2624 | <0.05 |
|  |  |  | Temperature | -0.0089 | 0.0070 | -0.3223 | -1.2643 | 0.2242 |
|  |  |  | Precipitation | -0.0016 | 0.0012 | -0.4945 | -1.3264 | 0.2033 |
|  |  |  | Relative humidity | 0.0018 | 0.0035 | 0.1426 | 0.5031 | 0.6218 |
|  |  |  | Evapotranspiration | -0.0005 | 0.0010 | -0.1515 | -0.5382 | 0.5978 |
|  |  |  | Elevation | -0.0001 | 0.0001 | -0.1553 | -0.7080 | 0.4891 |
|  | **SL** | 0.2449 | Intercept | 3.3935 | 0.3250 |  | 10.4420 | <0.05 |
|  |  |  | Temperature | -0.0007 | 0.0107 | -0.0207 | -0.0643 | 0.9496 |
|  |  |  | Precipitation | 0.0003 | 0.0018 | 0.0752 | 0.1596 | 0.8752 |
|  |  |  | Relative humidity | -0.0070 | 0.0054 | -0.4641 | -1.2954 | 0.2136 |
|  |  |  | Evapotranspiration | 0.0009 | 0.0015 | 0.2098 | 0.5896 | 0.5637 |
|  |  |  | Elevation | -0.0002 | 0.0001 | -0.5045 | -1.8201 | 0.0875 |
|  | **PW** | 0.5843 | Intercept | 2.4200 | 0.1365 |  | 17.7341 | <0.05 |
|  |  |  | **Temperature** | **-0.0123** | **0.0045** | **-0.6530** | **-2.7308** | **<0.05** |
|  |  |  | Precipitation | -0.0002 | 0.0008 | -0.0876 | -0.2506 | 0.8053 |
|  |  |  | Relative humidity | 0.0013 | 0.0023 | 0.1512 | 0.5690 | 0.5773 |
|  |  |  | Evapotranspiration | -0.0007 | 0.0006 | -0.2830 | -1.0719 | 0.2997 |
|  |  |  | Elevation | 0.0000 | 0.0001 | -0.1926 | -0.9364 | 0.3630 |
|  | **WL** | 0.3369 | Intercept | 4.4144 | 0.3605 |  | 12.2443 | <0.05 |
|  |  |  | Temperature | -0.0133 | 0.0119 | -0.3383 | -1.1201 | 0.2792 |
|  |  |  | Precipitation | -0.0022 | 0.0020 | -0.4853 | -1.0990 | 0.2880 |
|  |  |  | Relative humidity | 0.0018 | 0.0060 | 0.1004 | 0.2990 | 0.7688 |
|  |  |  | Evapotranspiration | 0.0005 | 0.0017 | 0.0906 | 0.2716 | 0.7894 |
|  |  |  | Elevation | -0.0001 | 0.0001 | -0.2254 | -0.8678 | 0.3983 |
|  | **ML** | 0.4765 | Intercept | 1.6921 | 0.2422 |  | 6.9873 | <0.05 |
|  |  |  | Temperature | -0.0063 | 0.0080 | -0.2130 | -0.7938 | 0.4389 |
|  |  |  | Precipitation | -0.0017 | 0.0014 | -0.4824 | -1.2294 | 0.2367 |
|  |  |  | Relative humidity | -0.0021 | 0.0040 | -0.1538 | -0.5155 | 0.6132 |
|  |  |  | Evapotranspiration | 0.0006 | 0.0011 | 0.1514 | 0.5111 | 0.6162 |
|  |  |  | Elevation | 0.0000 | 0.0001 | 0.0329 | 0.1426 | 0.8884 |

**Tab. S9:** Multiple linear regression estimates of trait means across worker subcaste and environmental variables. Significant values are highlighted in bold.

| **Worker subcaste** | **Trait** | **R^2^** | **Independent variable** | **Unstandardized coefficient** | | **Standardized coefficient** | ***t*** | ***P* value** |
| --- | --- | --- | --- | --- | --- | --- | --- | --- |
|  |  |  |  | **B** | **Std. error** | **Beta** |  |  |
| **Minor worker** | **HL** | 0.5983 | Intercept | 2.0703 | 0.1858 |  | 11.1420 | <0.05 |
|  |  |  | **Temperature** | **-0.0187** | **0.0061** | **-0.7190** | **-3.0588** | **<0.05** |
|  |  |  | **Precipitation** | **0.0032** | **0.0010** | **1.0559** | **3.0723** | **<0.05** |
|  |  |  | Relative humidity | -0.0049 | 0.0031 | 0.4165 | -1.5939 | 0.1305 |
|  |  |  | **Evapotranspiration** | **-0.0021** | **0.0009** | **0.6477** | **-2.4959** | **<0.05** |
|  |  |  | Elevation | 0.0001 | 0.0001 | 0.2017 | 0.9975 | 0.3334 |
|  | **HW** | 0.5485 | Intercept | 1.7958 | 0.1689 |  | 10.6340 | <0.05 |
|  |  |  | **Temperature** | **-0.0170** | **0.0056** | **-0.7610** | **-3.0534** | **<0.05** |
|  |  |  | **Precipitation** | **0.0027** | **0.0009** | **1.0400** | **2.8541** | **<0.05** |
|  |  |  | Relative humidity | -0.0052 | 0.0028 | -0.5103 | -1.8418 | 0.0841 |
|  |  |  | Evapotranspiration | -0.0014 | 0.0008 | -0.4830 | -1.7551 | 0.0984 |
|  |  |  | Elevation | 0.0000 | 0.0001 | 0.1034 | 0.4824 | 0.6361 |
|  | **SL** | 0.5604 | Intercept | 1.6873 | 0.2437 |  | 6.9247 | <0.05 |
|  |  |  | Temperature | 0.0128 | 0.0080 | 0.3919 | 1.5936 | 0.1306 |
|  |  |  | Precipitation | 0.0011 | 0.0014 | 0.2956 | 0.8221 | 0.4231 |
|  |  |  | Relative humidity | 0.0025 | 0.0041 | 0.1689 | 0.6178 | 0.5454 |
|  |  |  | Evapotranspiration | -0.0009 | 0.0011 | -0.2139 | -0.7880 | 0.4422 |
|  |  |  | Elevation | -0.0001 | 0.0001 | -0.1781 | -0.8422 | 0.4121 |
|  | **PW** | 0.3801 | Intercept | 1.6172 | 0.1942 |  | 8.3294 | <0.05 |
|  |  |  | Temperature | -0.0122 | 0.0064 | -0.5574 | -1.9088 | 0.0744 |
|  |  |  | Precipitation | 0.0015 | 0.0011 | 0.5927 | 1.3882 | 0.1841 |
|  |  |  | Relative humidity | -0.0051 | 0.0032 | -0.5161 | -1.5897 | 0.1315 |
|  |  |  | Evapotranspiration | -0.0007 | 0.0009 | -0.2466 | -0.7648 | 0.4555 |
|  |  |  | Elevation | 0.0000 | 0.0001 | -0.1320 | -0.5254 | 0.6065 |
|  | **WL** | 0.3937 | Intercept | 3.3263 | 0.2636 |  | 12.6176 | <0.05 |
|  |  |  | Temperature | -0.0156 | 0.0087 | -0.5167 | -1.7891 | 0.0925 |
|  |  |  | Precipitation | 0.0030 | 0.0015 | 0.8551 | 2.0250 | 0.0599 |
|  |  |  | Relative humidity | -0.0090 | 0.0044 | -0.6586 | -2.0514 | 0.0570 |
|  |  |  | Evapotranspiration | -0.0006 | 0.0012 | -0.1476 | -0.4629 | 0.6496 |
|  |  |  | Elevation | -0.0002 | 0.0001 | -0.4125 | -1.6608 | 0.1162 |
|  | **ML** | 0.0165 | Intercept | 0.5622 | 0.1875 |  | 2.9989 | <0.05 |
|  |  |  | Temperature | 0.0020 | 0.0062 | 0.1187 | 0.3227 | 0.7511 |
|  |  |  | Precipitation | 0.0000 | 0.0010 | -0.0240 | -0.0446 | 0.9650 |
|  |  |  | Relative humidity | 0.0009 | 0.0031 | 0.1225 | 0.2996 | 0.7683 |
|  |  |  | Evapotranspiration | -0.0002 | 0.0009 | -0.1090 | -0.2685 | 0.7917 |
|  |  |  | Elevation | 0.0000 | 0.0001 | 0.0504 | 0.1594 | 0.8753 |
